# Supplementary material for: Investigation on Intestinal Proteins and Drug Metabolizing Enzymes in Simulated Microgravity Rats by a Proteomics Method
Source: Molecules. 2020 Sep 24;25(19):4391. doi: 10.3390/molecules25194391 (PMC7582489; doi:10.3390/molecules25194391)
Supplement: Supplementary file 1 [file molecules-25-04391-s001.zip › Supplementary Table.pdf]

Supplemental Table The list of all differently expressed proteins

Gene IDs	Protein names	Fold change		*P*-value
O35787	Kinesin-like protein KIF1C	229.35	↑	0.0000
P61459	Pterin-4-alpha-carbinolamine dehydratase	22.90	↑	0.0207
Q07116	Sulfite oxidase, mitochondrial	14.37	↑	0.0003
Q9JM53	Apoptosis-inducing factor 1, mitochondrial	11.80	↑	0.0001
B2RYD2	Epithelial splicing regulatory protein 1	9.90	↑	0.0341
P08932	T-kininogen 2	9.60	↑	0.0000
P50554	4-aminobutyrate aminotransferase, mitochondrial	8.96	↑	0.0041
P09006	Serine protease inhibitor A3N	8.54	↑	0.0056
P02680	Fibrinogen gamma chain	8.28	↑	0.0337
P31430	Dipeptidase 1	8.10	↑	0.0384
Q9Z2G8	Nucleosome assembly protein 1-like 1	7.69	↑	0.0024
P13635	Ceruloplasmin	7.59	↑	0.0033
Q9Z1B2	Glutathione S-transferase Mu 5	7.54	↑	0.0092
P56522	NADPH:adrenodoxin oxidoreductase, mitochondrial	7.39	↑	0.0018
Q9EPF2	Cell surface glycoprotein MUC18	7.34	↑	0.0007
Q8CFN2	Cell division control protein 42 homolog	7.26	↑	0.0124
Q62940	E3 ubiquitin-protein ligase NEDD4	7.09	↑	0.0173
P18614	Integrin alpha-1	7.07	↑	0.0000
P68370	Tubulin alpha-1A chain	7.06	↑	0.0168
Q5HZE4	Methylthioribose-1-phosphate isomerase	6.84	↑	0.0156
P29066	Beta-arrestin-1	6.73	↑	0.0064
P55161	Nck-associated protein 1	6.47	↑	0.0011
P24329	Thiosulfate sulfurtransferase	6.41	↑	0.0001
P01048	T-kininogen 1	6.10	↑	0.0209

Continue Table

Gene IDs	Protein names	Fold change		*P*-value
Q9JHB5	Translin-associated protein X	5.88	↑	0.0030
P28826	Meprin A subunit beta	5.86	↑	0.0201
P14480	Fibrinogen beta chain	5.83	↑	0.0119
O54728	Phospholipase B1, membrane-associated	5.53	↑	0.0109
Q02253	Methylmalonate-semialdehyde dehydrogenase [acylating], mitochondrial	5.38	↑	0.0121
P07633	Propionyl-CoA carboxylase beta chain, mitochondrial	5.33	↑	0.0016
Q3B8Q2	Eukaryotic initiation factor 4A-III	5.30	↑	0.0016
P07872	Peroxisomal acyl-coenzyme A oxidase 1	5.21	↑	0.0443
P08011	Microsomal glutathione S-transferase 1	4.92	↑	0.0004
Q9Z1X1	Extended synaptotagmin-1	4.87	↑	0.0005
P25235	Dolichyl-diphosphooligosaccharide--protein glycosyltransferase subunit 2	4.86	↑	0.0015
P04644	40S ribosomal protein S17	4.81	↑	0.0121
Q62991	Sec1 family domain-containing protein 1	4.64	↑	0.0016
O35826	Bifunctional UDP-N-acetylglucosamine 2-epimerase/N-acetylmannosamine kinase	4.60	↑	0.0038
Q9JIL8	DNA repair protein RAD50	4.45	↑	0.0018
B0BNE2	DNA-directed RNA polymerases I, II, and III subunit RPABC1	4.45	↑	0.0187
B0BNM1	NAD(P)H-hydrate epimerase	4.41	↑	0.0197
B4F7E8	Niban-like protein 1	4.35	↑	0.0100
Q9JMJ4	Pre-mRNA-processing factor 19	4.24	↑	0.0178
Q3KR59	Ubiquitin carboxyl-terminal hydrolase 10	4.23	↑	0.0006
P01830	Thy-1 membrane glycoprotein	4.23	↑	0.0057
P62916	Transcription initiation factor IIB	4.18	↑	0.0489
P17077	60S ribosomal protein L9	4.04	↑	0.0011
Q9QYL8	Acyl-protein thioesterase 2	3.91	↑	0.0283

Continue Table

Gene IDs	Protein names	Fold change		*P*-value
Q07014	Tyrosine-protein kinase Lyn	3.91	↑	0.0076
Q99PF5	Far upstream element-binding protein 2	3.84	↑	0.0152
P62083	40S ribosomal protein S7 (S8)	3.81	↑	0.0167
Q5FVQ4	Malectin	3.76	↑	0.0008
P51952	Cyclin-dependent kinase 7	3.60	↑	0.0002
Q8K4C0	Dimethylaniline monooxygenase [N-oxide-forming] 5	3.57	↑	0.0071
Q6P756	Adaptin ear-binding coat-associated protein 2	3.52	↑	0.0060
Q56A18	SWI/SNF-related matrix-associated actin-dependent regulator of chromatin subfamily E member 1	3.51	↑	0.0034
Q9Z1L1	Claudin-7	3.50	↑	0.0168
B5DEH2	Erlin-2	3.49	↑	0.0356
B5DFC8	Eukaryotic translation initiation factor 3 subunit C	3.46	↑	0.0074
Q8K451	Probable G-protein coupled receptor 156	3.43	↑	0.0248
Q8K1Q0	Glycylpeptide N-tetradecanoyltransferase 1	3.37	↑	0.0498
Q8K4D8	Aldehyde dehydrogenase family 1 member A3	3.37	↑	0.0082
P63004	Platelet-activating factor acetylhydrolase IB subunit alpha	3.34	↑	0.0127
P19804	Nucleoside diphosphate kinase B	3.34	↑	0.0202
Q5FWY5	AH receptor-interacting protein	3.32	↑	0.0078
Q5RK19	Vacuolar-sorting protein SNF8	3.29	↑	0.0146
P81795	Eukaryotic translation initiation factor 2 subunit 3, X-linked	3.28	↑	0.0127
P18265	Glycogen synthase kinase-3 alpha	3.25	↑	0.0072
O70513	Galectin-3-binding protein	3.24	↑	0.0203
P00697	Lysozyme C-1	3.23	↑	0.0008
P50123	Glutamyl aminopeptidase	3.19	↑	0.0202
P07153	Dolichyl-diphosphooligosaccharide--protein glycosyltransferase subunit 1	3.17	↑	0.0000

Continue Table

Gene IDs	Protein names	Fold change		*P*-value
Q62636	Ras-related protein Rap-1b	3.15	↑	0.0004
P07340	Sodium/potassium-transporting ATPase subunit beta-1	3.15	↑	0.0468
P50398	Rab GDP dissociation inhibitor alpha	3.15	↑	0.0032
Q9EPB1	Dipeptidyl peptidase 2	3.11	↑	0.0001
Q5U216	ATP-dependent RNA helicase DDX39A	3.10	↑	0.0372
Q9JK11	Reticulon-4	3.09	↑	0.0098
P07379	Phosphoenolpyruvate carboxykinase, cytosolic	3.08	↑	0.0011
Q68FY0	Cytochrome b-c1 complex subunit 1, mitochondrial	3.05	↑	0.0046
P06866	Haptoglobin	3.03	↑	0.0354
Q8K3F4	Protein phosphatase 1 regulatory subunit 14D	3.01	↑	0.0013
P97834	COP9 signalosome complex subunit 1	2.97	↑	0.0013
P97840	Galectin-9	2.96	↑	0.0066
P49134	Integrin beta-1	2.91	↑	0.0312
P25031	Regenerating islet-derived protein 3-beta	2.90	↑	0.0020
P0C5E3	Palladin	2.89	↑	0.0259
O54772	SWI/SNF-related matrix-associated actin-dependent regulator of chromatin subfamily D member 2	2.89	↑	0.0000
P97690	Structural maintenance of chromosomes protein 3	2.86	↑	0.0173
Q9Z0V5	Peroxiredoxin-4	2.86	↑	0.0436
Q6XQN1	Nicotinate phosphoribosyltransferase	2.83	↑	0.0274
P32362	Uroporphyrinogen decarboxylase	2.81	↑	0.0041
O70257	Syntaxin-7	2.79	↑	0.0100
P43138	DNA-(apurinic or apyrimidinic site) lyase	2.78	↑	0.0015
Q6AYC2	Immunity-related GTPase family M protein	2.78	↑	0.0199
P85972	Vinculin	2.76	↑	0.0210

Continue Table

Gene IDs	Protein names	Fold change		*P*-value
P62718	60S ribosomal protein L18a	2.75	↑	0.0079
O89046	Coronin-1B	2.73	↑	0.0000
F1LM93	Tyrosine-protein kinase Yes	2.71	↑	0.0155
Q32PX7	Far upstream element-binding protein 1	2.71	↑	0.0122
Q6AXN3	Transmembrane emp24 domain-containing protein 5	2.70	↑	0.0131
P40112	Proteasome subunit beta type-3	2.70	↑	0.0018
P37996	ADP-ribosylation factor-like protein 3	2.69	↑	0.0485
P04762	Catalase	2.68	↑	0.0208
Q9EQP5	Prolargin	2.67	↑	0.0000
P48037	Annexin A6	2.67	↑	0.0056
P42676	Neurolysin, mitochondrial	2.66	↑	0.0464
P11507	Sarcoplasmic/endoplasmic reticulum calcium ATPase 2	2.65	↑	0.0024
Q9Z270	Vesicle-associated membrane protein-associated protein A	2.65	↑	0.0399
Q6P7Q1	BRISC and BRCA1-A complex member 2	2.64	↑	0.0001
P53042	Serine/threonine-protein phosphatase 5	2.64	↑	0.0224
Q07984	Translocon-associated protein subunit delta	2.63	↑	0.0088
P07896	Peroxisomal bifunctional enzyme	2.61	↑	0.0041
Q5M7A4	Ubiquitin-like modifier-activating enzyme 5	2.61	↑	0.0033
P13233	2',3'-cyclic-nucleotide 3'-phosphodiesterase	2.60	↑	0.0091
Q9Z1P2	Alpha-actinin-1	2.60	↑	0.0001
Q3KRD5	Mitochondrial import receptor subunit TOM34	2.60	↑	0.0007
B2GUV7	Eukaryotic translation initiation factor 5B	2.58	↑	0.0159
P17220	Proteasome subunit alpha type-2	2.58	↑	0.0184
P27881	Hexokinase-2	2.58	↑	0.0064

Continue Table

Gene IDs	Protein names	Fold change		*P*-value
Q9JID1	Programmed cell death protein 4	2.55	↑	0.0001
P14668	Annexin A5	2.55	↑	0.0067
P97571	Calpain-1 catalytic subunit	2.55	↑	0.0093
P01026	Complement C3	2.55	↑	0.0177
B0BNE5	S-formylglutathione hydrolase	2.53	↑	0.0011
D3ZBP4	[F-actin]-monooxygenase MICAL1	2.51	↑	0.0219
Q8VIF7	Methanethiol oxidase	2.51	↑	0.0000
P11497	Acetyl-CoA carboxylase 1	2.51	↑	0.0108
P04276	Vitamin D-binding protein	2.49	↑	0.0031
Q641Y0	Dolichyl-diphosphooligosaccharide--protein glycosyltransferase 48 kDa subunit	2.49	↑	0.0043
O08590	Membrane primary amine oxidase	2.49	↑	0.0107
Q4AE70	Histone-arginine methyltransferase CARM1	2.48	↑	0.0077
O70511	Ankyrin-3	2.48	↑	0.0172
Q5U1X1	Oligoribonuclease, mitochondrial	2.47	↑	0.0054
P97615	Thioredoxin, mitochondrial	2.47	↑	0.0020
P20069	Mitochondrial-processing peptidase subunit alpha	2.46	↑	0.0448
Q63862	Myosin-11	2.46	↑	0.0000
P17164	Tissue alpha-L-fucosidase	2.44	↑	0.0042
Q63663	Guanylate-binding protein 1	2.44	↑	0.0200
Q63081	Protein disulfide-isomerase A6	2.44	↑	0.0000
Q9QX79	Fetuin-B	2.43	↑	0.0033
Q63507	60S ribosomal protein L14	2.43	↑	0.0088
Q3T1J1	Eukaryotic translation initiation factor 5A-1	2.43	↑	0.0000
Q5I0E7	Transmembrane emp24 domain-containing protein 9	2.41	↑	0.0014

Continue Table

Gene IDs	Protein names	Fold change		*P*-value
P05708	Hexokinase-1	2.40	↑	0.0196
O55012	Phosphatidylinositol-binding clathrin assembly protein	2.40	↑	0.0182
P70645	Bleomycin hydrolase	2.40	↑	0.0091
P04041	Glutathione peroxidase 1	2.40	↑	0.0038
P11442	Clathrin heavy chain 1	2.39	↑	0.0174
Q63400	Claudin-3	2.33	↑	0.0025
Q66HG3	Beta-Ala-His dipeptidase	2.33	↑	0.0491
P11884	Aldehyde dehydrogenase, mitochondrial	2.33	↑	0.0075
Q63009	Protein arginine N-methyltransferase 1	2.32	↑	0.0001
P62268	40S ribosomal protein S23	2.30	↑	0.0355
Q6AYE2	Endophilin-B1	2.30	↑	0.0000
Q4KM65	Cleavage and polyadenylation specificity factor subunit 5	2.30	↑	0.0021
Q4KM49	Tyrosine--tRNA ligase, cytoplasmic	2.29	↑	0.0015
P29147	D-beta-hydroxybutyrate dehydrogenase, mitochondrial	2.28	↑	0.0003
P15178	Aspartate--tRNA ligase, cytoplasmic	2.28	↑	0.0136
Q923V8	Selenoprotein F	2.27	↑	0.0272
Q4QQW4	Histone deacetylase 1	2.27	↑	0.0020
Q9JJ22	Endoplasmic reticulum aminopeptidase 1	2.26	↑	0.0027
Q8CHN6	Sphingosine-1-phosphate lyase 1	2.26	↑	0.0421
Q66HS7	PDZ and LIM domain protein 3	2.26	↑	0.0020
P11598	Protein disulfide-isomerase A3	2.26	↑	0.0011
P68182	cAMP-dependent protein kinase catalytic subunit beta	2.25	↑	0.0076
G3V9R8	Heterogeneous nuclear ribonucleoprotein C	2.24	↑	0.0422
O08651	D-3-phosphoglycerate dehydrogenase	2.23	↑	0.0009

Continue Table

Gene IDs	Protein names	Fold change		*P*-value
P06685	Sodium/potassium-transporting ATPase subunit alpha-1	2.23	↑	0.0068
O35567	Bifunctional purine biosynthesis protein PURH	2.22	↑	0.0000
Q62639	GTP-binding protein Rheb	2.19	↑	0.0029
P30839	Fatty aldehyde dehydrogenase	2.17	↑	0.0188
P0DMW1	Heat shock 70 kDa protein 1B	2.17	↑	0.0010
P60123	RuvB-like 1	2.17	↑	0.0003
P63326	40S ribosomal protein S10	2.16	↑	0.0002
Q6P686	Osteoclast-stimulating factor 1	2.16	↑	0.0017
P54319	Phospholipase A-2-activating protein	2.15	↑	0.0364
Q62812	Myosin-9	2.15	↑	0.0430
P97536	Cullin-associated NEDD8-dissociated protein 1	2.14	↑	0.0406
Q5BK63	NADH dehydrogenase	2.14	↑	0.0426
Q9WU82	Catenin beta-1	2.09	↑	0.0053
P18418	Calreticulin	2.09	↑	0.0279
P63018	Heat shock cognate 71 kDa protein	2.08	↑	0.0011
P68255	14-3-3 protein theta	2.07	↑	0.0043
P00770	Mast cell protease 2	2.06	↑	0.0005
O35854	Branched-chain-amino-acid aminotransferase, mitochondrial	2.06	↑	0.0085
P00406	Cytochrome c oxidase subunit 2	2.06	↑	0.0043
P30904	Macrophage migration inhibitory factor	2.05	↑	0.0097
P28480	T-complex protein 1 subunit alpha	2.04	↑	0.0014
P15684	Aminopeptidase N	2.01	↑	0.0218
P38552	Galectin-4	0.50	↓	0.0008
P62198	26S proteasome regulatory subunit 8	0.50	↓	0.0005

Continue Table

Gene IDs	Protein names	Fold change		*P*-value
B0BNA5	Coactosin-like protein	0.50	↓	0.0064
P30835	ATP-dependent 6-phosphofructokinase, liver type	0.49	↓	0.0129
P29314	40S ribosomal protein S9	0.49	↓	0.0292
Q4V8A2	Cell division cycle protein 27 homolog	0.48	↓	0.0101
Q63468	Phosphoribosyl pyrophosphate synthase-associated protein 1	0.47	↓	0.0115
Q5U2R0	Methionine adenosyltransferase 2 subunit beta	0.47	↓	0.0238
Q9R1T3	Cathepsin Z	0.47	↓	0.0212
Q9JJW3	Up-regulated during skeletal muscle growth protein 5	0.46	↓	0.0205
P69736	Endothelial differentiation-related factor 1	0.46	↓	0.0103
P26772	10 kDa heat shock protein, mitochondrial	0.46	↓	0.0479
Q920P6	Adenosine deaminase	0.46	↓	0.0005
P02692	Fatty acid-binding protein, liver	0.46	↓	0.0078
P84100	60S ribosomal protein L19	0.46	↓	0.0047
Q7TP52	Carboxymethylenebutenolidase homolog	0.46	↓	0.0035
P11030	Acyl-CoA-binding protein	0.46	↓	0.0310
P11348	Dihydropteridine reductase	0.45	↓	0.0052
P11762	Galectin-1	0.45	↓	0.0066
P54313	Guanine nucleotide-binding protein G(I)/G(S)/G(T) subunit beta-2	0.44	↓	0.0056
P61314	60S ribosomal protein L15	0.44	↓	0.0029
Q6EV70	GDP-fucose protein O-fucosyltransferase 1	0.44	↓	0.0214
P55053	Fatty acid-binding protein, epidermal	0.44	↓	0.0033
Q6IG12	Keratin, type II cytoskeletal 7	0.44	↓	0.0165
O35165	Golgi SNAP receptor complex member 2	0.43	↓	0.0008
Q10728	Protein phosphatase 1 regulatory subunit 12A	0.43	↓	0.0002

Continue Table

Gene IDs	Protein names	Fold change		*P*-value
P62890	60S ribosomal protein L30	0.42	↓	0.0041
P62907	60S ribosomal protein L10a	0.42	↓	0.0224
P14562	Lysosome-associated membrane glycoprotein 1	0.42	↓	0.0067
Q4QQT3	CUGBP Elav-like family member 1	0.42	↓	0.0174
Q5XI29	Cleavage and polyadenylation specificity factor subunit 7	0.42	↓	0.0011
Q498R7	UPF0587 protein C1orf123 homolog	0.41	↓	0.0001
Q5QE78	Aldehyde oxidase 2	0.41	↓	0.0006
P12749	60S ribosomal protein L26	0.41	↓	0.0293
P62628	Dynein light chain roadblock-type 1	0.41	↓	0.0046
Q62871	Cytoplasmic dynein 1 intermediate chain 2	0.41	↓	0.0017
P63029	Translationally-controlled tumor protein	0.40	↓	0.0000
Q63768	Adapter molecule crk	0.40	↓	0.0144
P62161	Calmodulin-2	0.40	↓	0.0076
D3ZBN0	Histone H1.5	0.40	↓	0.0003
Q4FZU6	Annexin A8	0.39	↓	0.0057
P0C0R5	Phosphoinositide 3-kinase regulatory subunit 4	0.39	↓	0.0068
P23711	Heme oxygenase 2	0.39	↓	0.0018
P63025	Vesicle-associated membrane protein 3	0.39	↓	0.0097
Q6QI44	N-alpha-acetyltransferase 25, NatB auxiliary subunit	0.38	↓	0.0006
Q6MG49	Large proline-rich protein BAG6	0.38	↓	0.0004
P13601	Aldehyde dehydrogenase, cytosolic 1	0.38	↓	0.0005
Q62733	Lamina-associated polypeptide 2, isoform beta	0.37	↓	0.0481
Q5U211	Sorting nexin-3	0.37	↓	0.0046
P29410	Adenylate kinase 2, mitochondrial	0.37	↓	0.0021

Continue Table

Gene IDs	Protein names	Fold change		*P*-value
Q66H98	Caveolae-associated protein 2	0.37	↓	0.0022
P25809	Creatine kinase U-type, mitochondrial	0.37	↓	0.0014
P08426	Cationic trypsin-3	0.37	↓	0.0473
P02693	Fatty acid-binding protein, intestinal	0.36	↓	0.0007
P20650	Protein phosphatase 1A	0.35	↓	0.0434
P43244	Matrin-3	0.34	↓	0.0005
P49911	Acidic leucine-rich nuclear phosphoprotein 32 family member A	0.34	↓	0.0138
P20037	Rano class II histocompatibility antigen, B alpha chain	0.34	↓	0.0000
Q08415	Kynurenine--oxoglutarate transaminase 1, mitochondrial	0.34	↓	0.0003
Q99MZ8	LIM and SH3 domain protein 1	0.34	↓	0.0143
G3V7P1	Syntaxin-12	0.34	↓	0.0005
Q01177	Plasminogen	0.33	↓	0.0028
Q62736	Non-muscle caldesmon	0.33	↓	0.0074
Q64303	Serine/threonine-protein kinase PAK 2	0.32	↓	0.0066
P14942	Glutathione S-transferase alpha-4	0.32	↓	0.0041
Q9QZR6	Septin-9	0.32	↓	0.0194
P70470	Acyl-protein thioesterase 1	0.31	↓	0.0249
O35831	Cyclin-dependent kinase 17	0.31	↓	0.0002
Q2LAP6	Testin	0.31	↓	0.0104
P81799	N-acetyl-D-glucosamine kinase	0.31	↓	0.0030
Q925G0	RNA-binding protein 3	0.31	↓	0.0235
P00774	Chymotrypsin-like elastase family member 2A	0.30	↓	0.0140
P11915	Non-specific lipid-transfer protein	0.30	↓	0.0089
P62870	Elongin-B	0.30	↓	0.0353

Continue Table

Gene IDs	Protein names	Fold change		*P*-value
Q568Z6	IST1 homolog	0.30	↓	0.0089
Q6PCU2	V-type proton ATPase subunit E 1	0.29	↓	0.0012
P00388	NADPH--cytochrome P450 reductase	0.28	↓	0.0122
P60868	40S ribosomal protein S20	0.28	↓	0.0141
P60892	Ribose-phosphate pyrophosphokinase 1	0.27	↓	0.0158
Q9QXU8	Cytoplasmic dynein 1 light intermediate chain 1	0.27	↓	0.0326
Q5U2R7	LRP chaperone MESD	0.27	↓	0.0283
Q9JJ19	Na(+)/H(+) exchange regulatory cofactor NHE-RF1	0.27	↓	0.0007
P04639	Apolipoprotein A-I	0.26	↓	0.0035
Q5RJP0	Aldose reductase-related protein 1	0.26	↓	0.0044
Q5XIU5	Proteasome inhibitor PI31 subunit	0.25	↓	0.0031
Q8CJD3	Zymogen granule membrane protein 16	0.25	↓	0.0113
Q6AY63	ADP-sugar pyrophosphatase	0.25	↓	0.0003
G3V6S8	Serine/arginine-rich splicing factor 6	0.25	↓	0.0398
O88637	Ethanolamine-phosphate cytidylyltransferase	0.24	↓	0.0067
Q6PEC1	Tubulin-specific chaperone A	0.24	↓	0.0031
O88794	Pyridoxine-5'-phosphate oxidase	0.24	↓	0.0111
P00689	Pancreatic alpha-amylase	0.24	↓	0.0425
Q9Z0W7	Chloride intracellular channel protein 4	0.24	↓	0.0007
Q5RK30	Ribosome maturation protein SBDS	0.24	↓	0.0273
P14046	Alpha-1-inhibitor 3	0.24	↓	0.0020
Q01750	General transcription factor IIF subunit 2	0.23	↓	0.0052
Q01986	Dual specificity mitogen-activated protein kinase kinase 1	0.23	↓	0.0001
O35094	Mitochondrial import inner membrane translocase subunit TIM44	0.23	↓	0.0000

Continue Table

Gene IDs	Protein names	Fold change		*P*-value
Q6AYR2	Protein NDRG3	0.23	↓	0.0011
P35434	ATP synthase subunit delta, mitochondrial	0.23	↓	0.0039
P24268	Cathepsin D	0.22	↓	0.0002
Q80Z29	Nicotinamide phosphoribosyltransferase	0.22	↓	0.0101
Q9WUC4	Copper transport protein ATOX1	0.22	↓	0.0014
P04903	Glutathione S-transferase alpha-2	0.21	↓	0.0002
Q71UE8	NEDD8	0.21	↓	0.0080
P05765	40S ribosomal protein S21	0.21	↓	0.0099
Q62952	Dihydropyrimidinase-related protein 3	0.21	↓	0.0236
P62845	40S ribosomal protein S15	0.21	↓	0.0244
P08699	Galectin-3	0.21	↓	0.0140
P09895	60S ribosomal protein L5	0.21	↓	0.0001
P06768	Retinol-binding protein 2	0.20	↓	0.0213
P00884	Fructose-bisphosphate aldolase B	0.20	↓	0.0464
P19511	ATP synthase F(0) complex subunit B1, mitochondrial	0.20	↓	0.0322
B2GV24	E3 UFM1-protein ligase 1	0.20	↓	0.0003
P27139	Carbonic anhydrase 2	0.19	↓	0.0000
Q5EGY4	Synaptobrevin homolog YKT6	0.19	↓	0.0184
P28042	Single-stranded DNA-binding protein, mitochondrial	0.18	↓	0.0346
Q9Z1N4	3'(2'),5'-bisphosphate nucleotidase 1	0.18	↓	0.0111
Q8K1P7	Transcription activator BRG1	0.18	↓	0.0042
P61751	ADP-ribosylation factor 4	0.17	↓	0.0025
O54980	Synaptogyrin-2	0.17	↓	0.0044
P17078	60S ribosomal protein L35	0.17	↓	0.0007

Continue Table

Gene IDs	Protein names	Fold change		*P*-value
O54735	cGMP-specific 3',5'-cyclic phosphodiesterase	0.17	↓	0.0001
P52873	Pyruvate carboxylase, mitochondrial	0.16	↓	0.0088
P02767	Transthyretin	0.16	↓	0.0056
P52925	High mobility group protein B2	0.16	↓	0.0482
P04961	Proliferating cell nuclear antigen	0.15	↓	0.0001
P49242	40S ribosomal protein S3a	0.14	↓	0.0187
P46418	Glutathione S-transferase alpha-5	0.14	↓	0.0345
Q3B7D1	Ubiquitin-conjugating enzyme E2 Z	0.12	↓	0.0011
P62282	40S ribosomal protein S11	0.12	↓	0.0034
P02651	Apolipoprotein A-IV	0.12	↓	0.0200
P06757	Alcohol dehydrogenase 1	0.12	↓	0.0132
Q497C3	Methyltransferase-like 26	0.12	↓	0.0265
P52847	Sulfotransferase family cytosolic 1B member 1	0.09	↓	0.0197
P83868	Prostaglandin E synthase 3	0.09	↓	0.0006
P11250	60S ribosomal protein L34	0.08	↓	0.0000
P19132	Ferritin heavy chain	0.08	↓	0.0219
F1LNI5	Protein phosphatase 1G	0.07	↓	0.0003
Q99MS0	SEC14-like protein 2	0.07	↓	0.0230
P84083	ADP-ribosylation factor 5	0.06	↓	0.0022
D4A4T9	Cysteine and histidine-rich domain-containing protein 1	0.06	↓	0.0192
Q6AYC8	SH2 domain-containing protein 4A	0.05	↓	0.0064
Q01984	Histamine N-methyltransferase	0.05	↓	0.0017
Q5XI97	Alanyl-tRNA editing protein Aarsd1	0.02	↓	0.0172
