# Supplementary material for: Investigation on Intestinal Proteins and Drug Metabolizing Enzymes in Simulated Microgravity Rats by a Proteomics Method
Source: Molecules. 2020 Sep 24;25(19):4391. doi: 10.3390/molecules25194391 (PMC7582489; doi:10.3390/molecules25194391)
Supplement: Supplementary file 1 [file molecules-25-04391-s001.zip › Supplementary Figure.pdf]

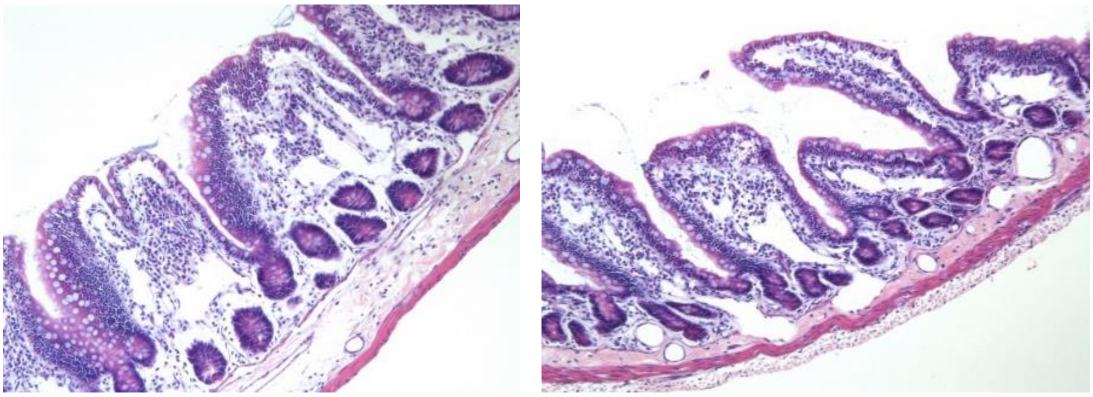

CON SMG

Supplemental Fig.1 The histomorphology of rat intestine under 14d SMG. The small intestinal villi of 14d SMG rats was abnormal in morphology with signs of necrosis and exfoliation. The crypts were swollen and the number of goblet cells in the crypt was reduced.

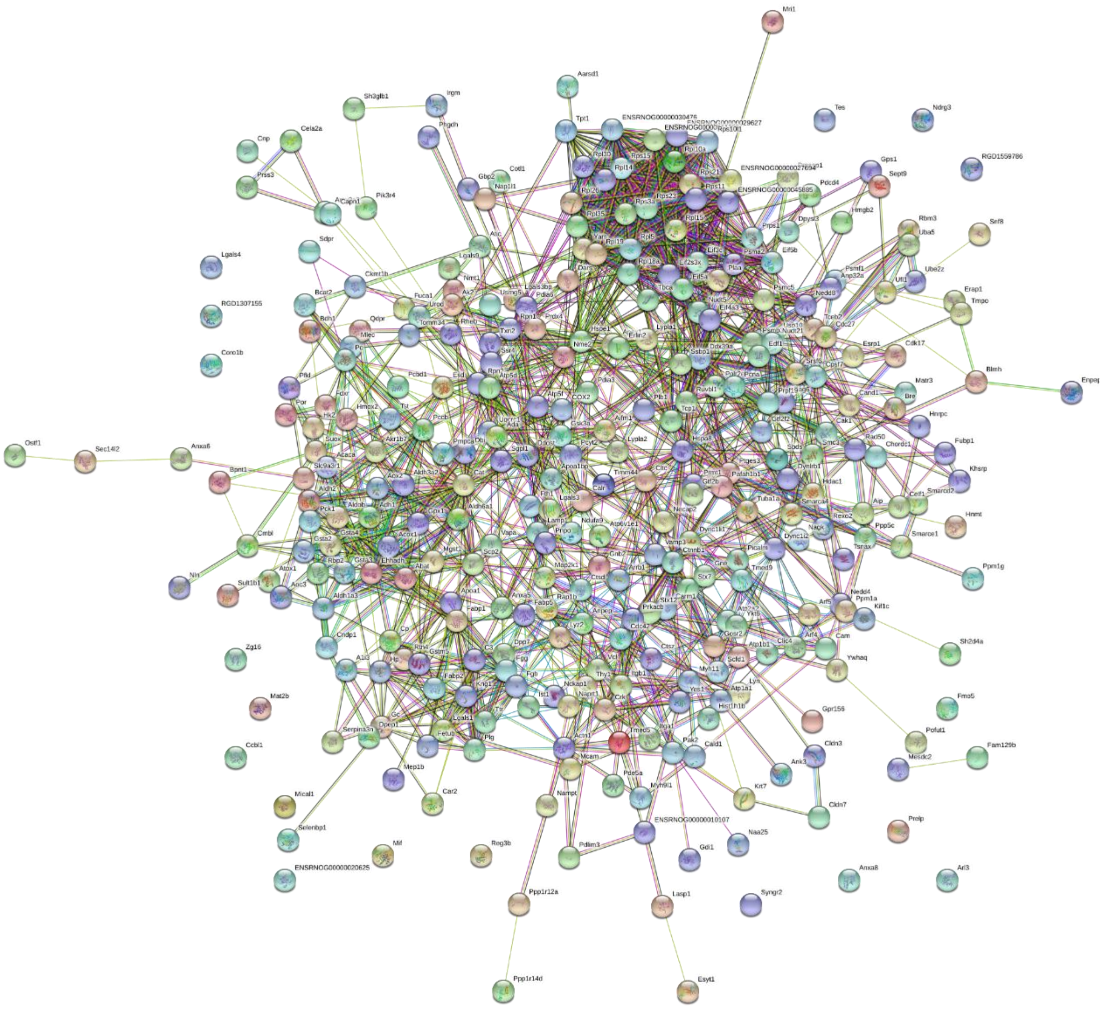

Supplemental Fig. 2 The protein-protein interaction network of all differentially expressed proteins (DEPs). The DEPs formed a complex protein-protein interaction network, and some of them could directly interact with other several proteins. Direct or indirect interaction of DEPs may affect potentially some pathways.
